# Simulation-Based Research on Phytoconstituents of *Embelia ribes* Targeting Proteins with Pathophysiological Implications in Rheumatoid Arthritis

**DOI:** 10.3390/life13071467

**Published:** 2023-06-28

**Authors:** Andrei-Flavius Radu, Paul Andrei Negru, Ada Radu, Alexandra Georgiana Tarce, Simona Gabriela Bungau, Mihaela Alexandra Bogdan, Delia Mirela Tit, Bogdan Uivaraseanu

**Affiliations:** 1 Doctoral School of Biological and Biomedical Sciences, University of Oradea, 410087 Oradea, Romania; andreiflavius.radu@uoradea.ro (A.-F.R.); dtit@uoradea.ro (D.M.T.); uivaraseanu_bogdan@yahoo.com (B.U.); 2 Department of Preclinical Disciplines, Faculty of Medicine and Pharmacy, University of Oradea, 410073 Oradea, Romania; 3 Ducfarm Pharmacy, 410514 Oradea, Romania; adaroman96@gmail.com; 4 Medicine Program of Study, Faculty of Medicine and Pharmacy, University of Oradea, 410073 Oradea, Romania; tarce_alexandra@yahoo.com; 5 Department of Pharmacy, Faculty of Medicine and Pharmacy, University of Oradea, 410028 Oradea, Romania; mabogdan@csud.uoradea.ro; 6 Department of Surgery Disciplines, Faculty of Medicine and Pharmacy, University of Oradea, 410073 Oradea, Romania

**Keywords:** rheumatoid arthritis, *Embelia ribes*, molecular docking, virtual screening, embelin, phytochemistry

## Abstract

Rheumatoid arthritis (RA) is a heterogeneous inflammatory disease with an autoimmune origin and an incompletely elucidated pathophysiological mechanism. RA pharmacotherapy is based on chemically or biologically active substances that provide clinical alleviation and remission, but the disease is still incurable. As a result, there remains a need for significant therapeutic development, and adjuvant therapies may play an essential role in the search for novel RA treatment strategies. The aim of the present study was to investigate potential phytocompounds and phytocompound derivates as RA treatment agents, using in silico methodologies. In this regard, five phytoconstituents identified in different structures of *Embelia ribes* were evaluated by in silico methods for their potential action on target proteins of therapeutic interest in RA. The methodology involved identifying the phytocompound with the highest binding toward the target protein via molecular docking using AutoDock Vina 1.5.7, followed by a ligand-based virtual screening based on the structure of the most promising phytocompound using SwissSimilarity. This process led to the identification of ligands that are not currently utilized in medical practice, but that might have the potential to be used in the management of RA after further extensive experimental endorsements. ZINC000004024651 showed the highest binding affinity for the Bruton’s tyrosine kinase protein, followed by ZINC000000434197 for p38 mitogen-activated protein kinases, ZINC000087606977 for interleukin-1 receptor-associated kinase 4, and ZINC000014728393 for matrix metallopeptidase 9, the latter two showing higher affinity than the co-crystallized compound. The relatively high affinities to target proteins and the pharmacokinetic data obtained by in silico studies using SwisADME suggest a first step for the inclusion of promising new compounds in various more advanced studies, leading to the evaluation of efficacy and safety profiles.

## 1. Introduction

Inflamed joints with degenerating cartilage and bone are indicators of rheumatoid arthritis (RA). RA is defined by extra-articular manifestations and increasing articular impairment, which can result in permanent incapacity, and is correlated with a high death rate. RA is the most common autoimmune inflammatory disorder among rheumatic degenerative musculoskeletal disorders [1,2]. 

Clinically, the signs and manifestations of RA are vastly different between the disorder’s early stages (i.e., swollen joints, fatigue, etc.) and its later, inadequately treated phases. Poorly managed RA exhibits a complicated clinical scenario, with the emergence of severe underlying manifestations such as lung nodules, lymphomas, hematologic disorders, and atherosclerosis [3]. 

Genetic factors (dysregulated citrullination, dysfunction of human leukocyte antigen-DR1 and human leukocyte antigen-DR4 genes) and environmental factors (air pollution, smoking, infections, inflammatory diet, etc.) contribute to the multifactorial nature of RA. Scientific research has suggested that there is a 50% genetic risk for RA [4].

The activation and mobilization of interleukin-1 (IL-1), interleukin-6 (IL-6), and tumor necrosis factor-α (TNF-α) have also been linked to the pathophysiology of RA by disrupting immunological homeostasis. Furthermore, TNF-α and IL-1 stimulate synovial cell production of matrix metalloproteases that degrade tissue, and, additionally, TNF-α promotes osteoclast formation. Additionally, the infiltration of synovium by osteoclasts and synoviocytes is triggered by the influx of T cells, macrophages, fibrocytes, and B cells in inflammatory joints [5].

In order to evaluate various types of drugs used in the treatment of RA, researchers and clinicians have conducted a significant number of clinical studies to date [6,7].

The target of the latest developments in the pharmacotherapeutic approach to RA is symptom improvement, delaying the progression of RA, and, eventually, complete remission. These developments are centered on the ongoing advancement of drug technology innovations [8]. Two different types of pharmacological management for RA are proposed as strategies: a symptomatic approach, with glucocorticoids and nonsteroidal anti-inflammatory drugs, and a more specific strategy using disease-modifying antirheumatic drugs, which can be conventional synthetic, targeted synthetic, or biological [9,10].

Even though mechanistic and therapeutic advancements have made remission possible, there is still an unmet need to completely cure this autoimmune illness. Consequently, considerable pharmacotherapeutic management development is still required, and adjuvant therapy may be an important element in the search for new approaches to RA [11].

Based on their traditional applications, biocompounds from plants have been widely investigated for potential implementation in medical strategies [12]. Plant bioactive product research has advanced, and the anti-inflammatory potential of various species has been evaluated, with promising results [13,14]. Alternative therapies have become more influential as viable therapies for chronic diseases because of the considerable negative impact that allopathic medicines may have as side effects [15]. 

Plants’ anti-inflammatory effects due to specific phytoconstituents might potentially offer management options for RA. Among them is the species *Embelia ribes*, a branching plant from the Myrsinaceae family. It possesses biological activity on a variety of pathways, with the anti-inflammatory effect being one of the most promising. Various parts of the plant are correlated with the species’ phytochemical composition. Embelin, embelinol, rapanone, catechin, and sitosterol are some of the most important phytoconstituents, due to their potential anti-inflammatory and antioxidant effects [16].

Embelin, a benzoquinone, and embelinol, an alcohol, are both found in the seeds of the *Embelia ribes* plant [16,17]. During research on the entire plant, a benzoquinone called rapanone was discovered [18]. Furthermore, along with sitosterol, catechin is a flavan-3-ol that was extracted from the roots’ ethanolic extract [19]. 

Embelin is the most promising bioactive compound from *Embelia ribes*; it has been shown to have the capacity to reduce cyclooxygenase-2 synthesis and protein kinase B activation in experimental investigations. The function of matrix metalloproteinase was reduced in cells that had been exposed to embelin [20]. The possibility of developing novel drugs starting with bioactive chemicals from several possible plants has been thoroughly examined in in silico studies [21,22]. Due to the fact that RA is a complex disease with an incompletely elucidated pathophysiological mechanism, there is no single well-characterized target for therapeutic agents. The scientific literature contains numerous studies using various approaches, including in vivo, in vitro, and in silico methods, to explore potential therapeutic targets for RA and identify potential new active compounds. The exploration of natural compounds as possible therapeutic options is widely used, as these compounds are generally expected to have fewer adverse reactions. In silico studies, and molecular docking in particular, have been used to evaluate the potential interaction and potential effects of ginsenosides in targeting the BTK and p38 MAPK signaling pathways, suggesting potential benefits for RA [23,24]. Another in silico study that also used molecular docking showed that compounds from *Hedyotis diffusa* Willd (i.e., β-sitosterol, quercetin, stigmasterol, kaempferol, 2-methoxy-3-methyl-9,10-anthraquinone) may have a therapeutic effect on RA by modulating the phosphatidylinositol 3-kinase/protein kinase B signaling pathways and targeting TNF-α, IL6, IL-, and target transcription factor p65 [25]. Moreover, other in silico studies evaluating the potential of natural compounds in inflammatory pathologies have identified possible actions on Toll-like receptor 4/activator protein 1 [26], prostaglandin-endoperoxide synthase 2 and caspase 3, molecules with a relevant role in RA pathophysiology [27].

In addition to in silico studies, in vitro and in vivo studies have contributed to expanding information on the characterization of RA. One such study explored the in vivo and in vitro effects of *Ribes orientale* against RA. Sprague-Dawley rats were subjected to arthritis induction through immunization. The extract and fractions effectively suppressed paw swelling and arthritic scores and prevented cachexia. Radiographic and histopathological evaluations confirmed the positive impact on joint architecture. The plant exhibited antioxidant activity, and phytochemical analysis identified the presence of flavonoids and polyphenols. The study also performed in vitro analysis by analyzing the inhibition of protein denaturation by egg albumin and bovine serum albumin. The *Ribes orientale* extract showed potent anti-denaturation and membrane-stabilizing effects [28].

Another study that focused on exploring the use of natural compounds in RA conducted a series of in vitro explorations on bioactive compounds from *Dodonaea viscosa*. In vitro 1,1-Diphenyl-2-picrylhydrazyl free radical scavenging activity and ferric reducing antioxidant power assays demonstrated that the plant extract had substantial antioxidant properties. The anti-inflammatory effects were demonstrated by performing protein denaturation and human red blood cell membrane stabilization assays. The findings suggest that active compounds from *Dodonaea viscosa* (i.e., phenols, flavonoids, steroids, sterols, saponins, coumarins, tannins, and terpenoids) could be successfully used in RA [29].

The management of RA may be affected by the promising candidates matrix metallopeptidase 9 (MMP-9), Bruton’s tyrosine kinase (BTK), IL-1 receptor-associated kinase 4 (IRAK4) [30], and p38 mitogen-activated protein kinases (p38 MAPK) [31]. 

Tissue remodeling and extracellular matrix degradation are linked to MMP-9 regulation. In RA, MMP-9 overexpression damages joint tissues. Its inhibition may prevent RA joint deterioration by reducing cartilage and bone damage [32]. B cell receptor signaling involves the enzyme BTK. BTK plays a key role in the activation and maturation of B cells, which lead to the production of autoantibodies in RA. BTK inhibition may reduce the RA-related autoimmune response and inflammation by inhibiting B cell activation and antibody generation [33]. IRAK-4 is a signaling molecule implicated in the Toll-like receptor and IL-1R signaling pathways. These mechanisms are critical to RA inflammation and immunological responses. Inhibiting IRAK-4 can reduce the inflammatory response in RA by modulating the TLR/IL-1R signaling cascade [34]. Furthermore, p38 MAPK regulates pro-inflammatory cytokines, such as TNF-α, IL-1, and IL-6, which are significant cytokines for RA inflammation. Inhibiting p38 MAPK reduces pro-inflammatory cytokines and RA symptoms [35].

Based on their modulatory role in RA pathophysiology and the correlation between their overexpression and bone and tissue damage, it is essential to identify and preliminarily evaluate, based on molecular docking studies, ligand-based virtual screening, and pharmacokinetic assessments, compounds with the potential to interact with these key enzymes. Simulations of interactions between ligands and target proteins lead to the evaluation of compounds that may have potential for use in advanced phases of the search (i.e., molecular dynamics, in vitro and in vivo studies) for new molecules with the potential to inhibit these key enzymes.

The aim of the present research was to apply methods of in silico studies (i.e., molecular docking, virtual screening, and pharmacokinetic data evaluation) in order to highlight new compounds without current medical use and with anti-inflammatory and antioxidant potential, starting from ligands with biological action demonstrated in the literature and ending with target proteins studied as having implications in several pathways of RA pathophysiology. The contribution to the current state of knowledge is based on the need for new chemical, biological, and phytochemical options to improve the control of RA in patients who are unresponsive to treatment, and the novelty of the approach is based on the selection of ligands identified in *Embelia ribes*, a species not previously addressed by computational approaches.

## 2. Results and Discussions

### 2.1. Molecular Docking Strategy Targeting BTK

In order to validate the molecular docking method, re-docking of the compound with which the protein co-crystallizes is performed. For both validation and docking, the grid box size was set to 60 × 60 × 60. It was given the coordinates X = 17.00, Y = 6.72, and Z = 15.09. The root-mean-square deviation (RMSD) should be less than 2 Å and within the accepted limits in the literature. The protein BTK is co-crystallized with pyrolopyrimidine, 7-Cyclopentyl-5-(4-phenoxyphenyl)-7H-pyrrolo [2,3-d] pyrimidin-4-ylamine.

**Figure 1:**
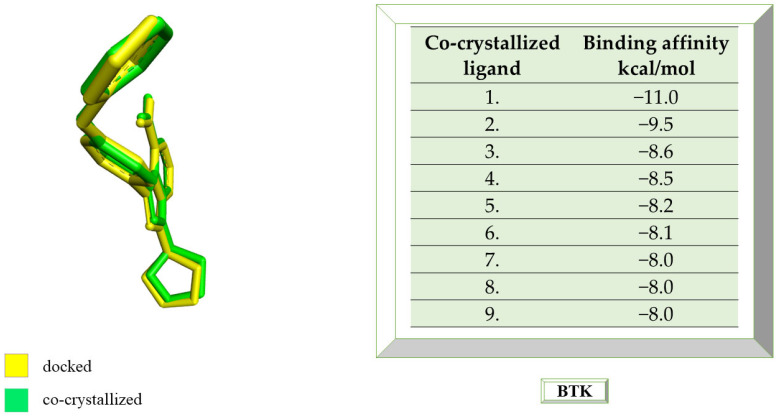
The highest binding affinity values for BTK of the re-docked native ligand, and the structure of the native ligand overlapped with the structure of the re-docked ligand. BTK, Bruton’s tyrosine kinase.

The RMSD value calculated with AutoDock Tools is 0.78 Å, indicating a proper molecular docking process that predicts, with high accuracy, the ligand-protein interaction. The natively re-docked ligand showed an affinity of −11 kcal/mol, and, in Figure 1, the docked structure is superimposed on the co-crystallized one.

The ligands in the study were docked to determine their binding affinity for the protein and to determine which one has the highest potential to interact with BTK. Table 1 shows the affinities of the most stable conformations to the protein.

Of the compounds studied, catechin and sitosterol show the highest affinity for BTK. Rapanone has the lowest affinity of the five ligands studied.

Through non-covalent interactions, including van der Waals forces, electrostatic interactions, hydrogen bonds, and hydrophobic interactions, ligands interact with target molecules in molecular docking research. A ligand’s optimal direction and conformation within the protein binding site are predicted using a docking simulation, which optimizes the positive interactions and minimizes the unfavorable ones between the ligand and the target protein [36].

**Figure 2:**
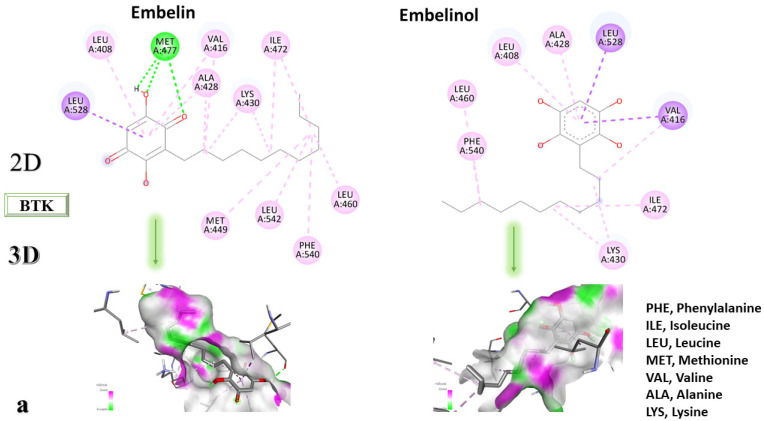

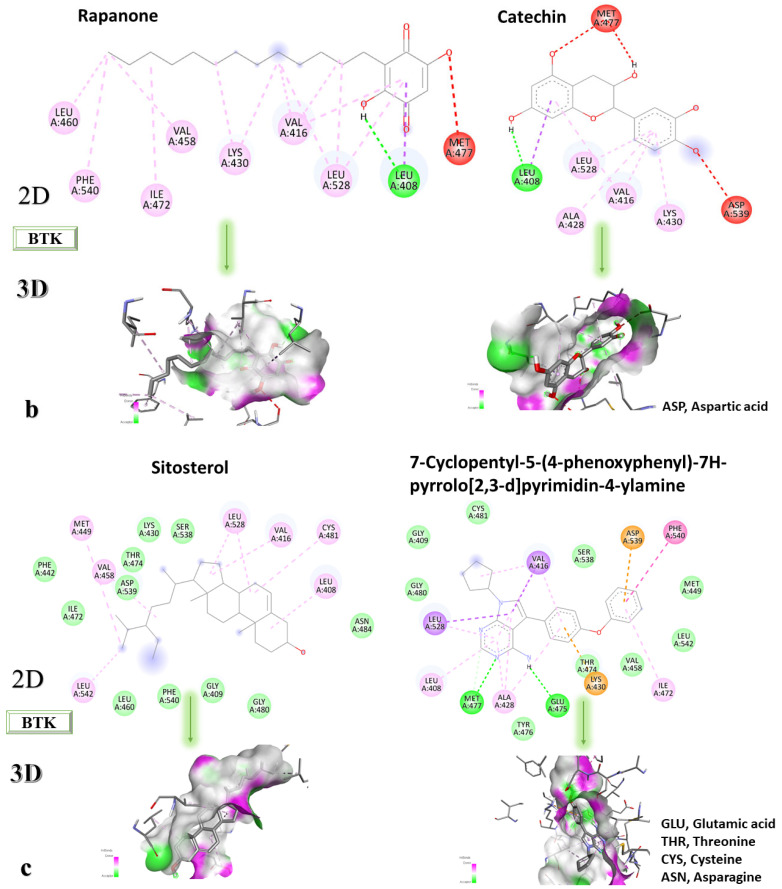
In-depth 2D and 3D interactions of ligands with different BTK constituent amino acids. BTK, Bruton’s tyrosine kinase. (**a**). Interactions between the chemical structures of embelin and embelinol with the amino acids of BTK; (**b**). Interactions between the chemical structures of rapanone and catechin with the amino acids of BTK; (**c**). Interactions between the chemical structures of sitosterol and 7-Cyclopentyl-5-(4-phenoxyphenyl)-7H-pyrrolo[2,3-d]pyrimidin-4-ylamine with the amino acids of BTK.

Figure 2a–c shows 2D diagrams representing the interactions between the studied ligands and the protein, and 3D diagrams showing the ligands in the binding pocket of the protein.

According to the 2D diagram, embelin interacts with MET477, LEU408, LEU528, MET449, LEU542, LEU540, LEU460, ILE472, LYS430, ALA428, and VAL415. Moreover, embelin forms hydrogen bonds with MET499. Embelinol interacts with VAL416, LEU528, ALA428, LEU408, LEU460, PHE540, LYS430, and ILE472. Rapanone interacts with MET477, LEU408, LEU528, VAL416, LYS430, VAL458, ILE472, PHE540, and LEU560, of which the bond with LEU408 is of the conventional hydrogen bond type. Catechin binds to the following amino acids: MET477, LEU408, LEU528, ALA428, VAL416, LYS430, and ASP539, of which the conventional hydrogen bond is formed with LEU408. Sitosterol, the final ligand tested, has the highest affinity for BTK of the docked compounds, and interacts with LEU408, CYS481, VAL416, LEU528, VAL 458, MET449, and LEU542. The re-docked compound interacts with PHE540, ASP539, VAL416, LEU528, LEU408, MET477, ALA428, GLU475, LYS430, and ILE472, of which conventional hydrogen bonds are formed with MET477 and GLU475.

### 2.2. Molecular Docking Strategy Targeting p38 MAPK

**Figure 3:**
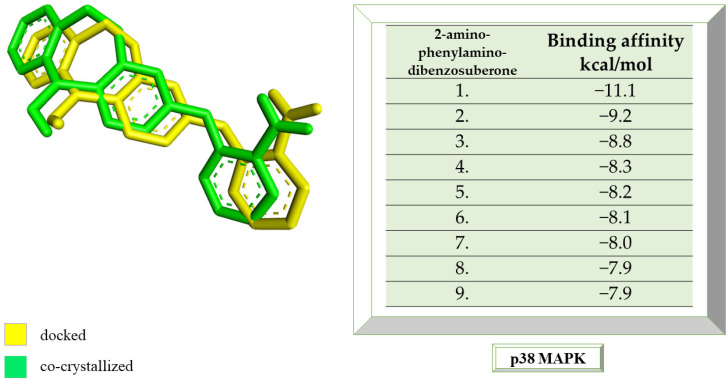
The highest binding affinity values for p38 MAPK of the re-docked native ligand, and the structure of the native ligand overlapped with the structure of the re-docked ligand. p38 MAPK, p38 mitogen-activated protein kinases.

As with the BTK interaction assessments, the compound with which the protein co-crystallizes was re-docked to determine the RMSD value. The grid box value remains 60 × 60 × 60, and the coordinates are set to X = 12.90, Y = 15.41, Z = 31.36. p38 MAPK comes co-crystallized with 2-amino-phenylamino-dibenzosuberone. The RMSD value of 1.23 Å indicates a proper molecular docking process that predicts, with high accuracy, the ligand-protein interaction. Following re-docking, the co-crystallized compound showed an affinity of −11.1 kcal/mol towards the target protein. In Figure 3, the structure of the docked native ligand is overlaid with the structure of the co-crystallized ligand, and the affinities of the most stable conformations towards p38 MAPK are depicted.

The binding affinities of the ligands studied for their potential to interact with p38 MAPK are shown in Table 2.

**Figure 4:**
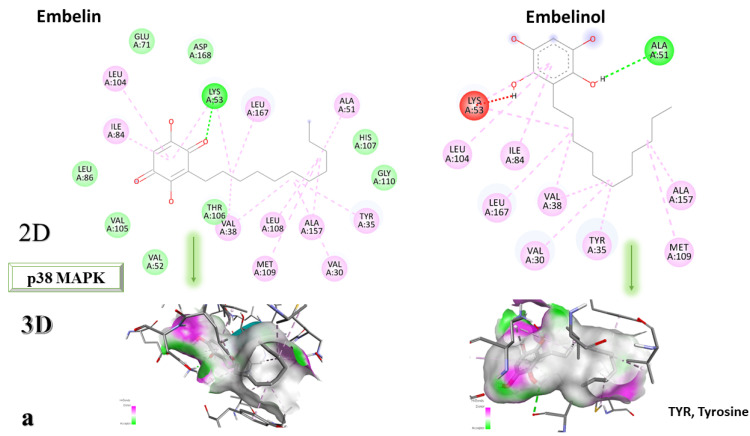

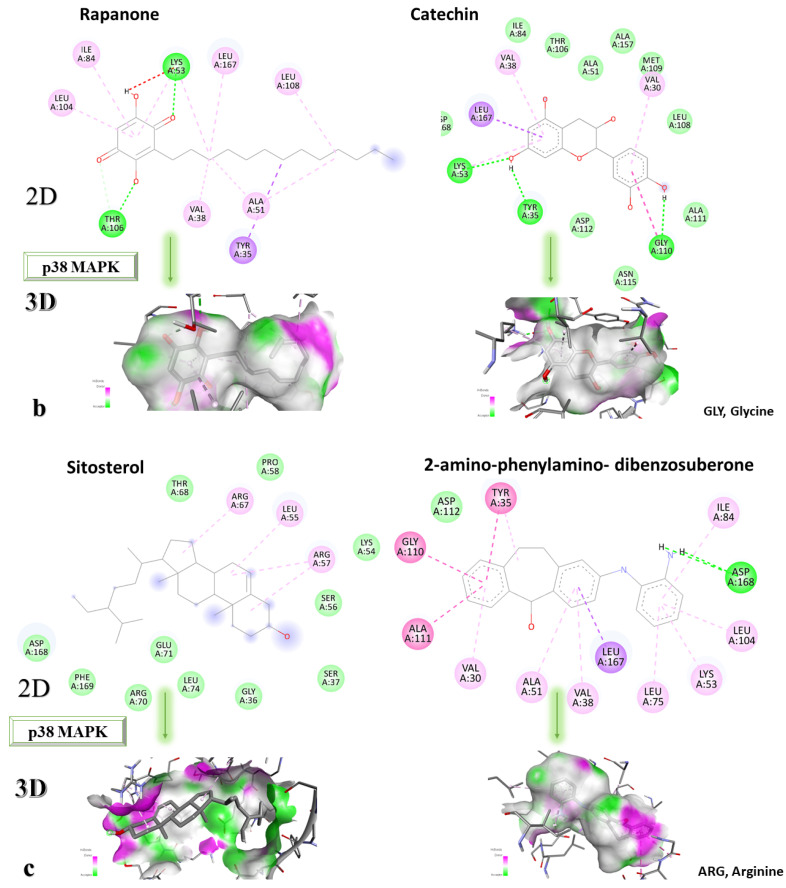
In-depth 2D and 3D interactions of ligands with different p38 MAPK constituent amino acids. p38 MAPK, p38 mitogen-activated protein kinases. (**a**). Interactions between the chemical structures of embelin and embelinol with the amino acids of p38 MAPK; (**b**). Interactions between the chemical structures of rapanone and catechin with the amino acids of p38 MAPK; (**c**). Interactions between the chemical structures of sitosterol and 2-amino-phenylamino-dibenzosuberone with the amino acids of p38 MAPK.

From the compounds studied, embelin is the compound with the highest binding affinity for p38 MAPK, with −8.4 kcal/mol, followed by catechin, with −8.2 kcal/mol. The compound with the lowest protein affinity is embelinol, with −7.7 kcal/mol. Figure 4a–c depicts the type of ligand-protein binding in both 2D and 3D formats.

From the analysis of the 2D diagrams, it can be observed that embelin interacts with ALA51, LEU167, LYS53, LEU104, ILE84, VAL38, LEU108, MET109, ALA157, VAL30, and TYR35, with which it forms conventional hydrogen bonds with LYS53. Embelinol interacts with ALA51, LYS53, LEU104, ILE84, LEU167, VAL38, VAL30, TYR35, MET109, and ALA157, with which it forms conventional hydrogen bonds with ALA51. Rapanone interacts with LEU108, LEU167, LYS53, ILE84, LEU104, THR106, VAL38, ALA51, and TYR35, of which LYS53 and THR106 form conventional hydrogen bonds. Catechin interacts with VAL30, VAL38, LEU167, LYS53, TYR35, and GLY110, forming conventional hydrogen bonds with LYS53, TYR35, and GLY110. Sitosterol interacts with ARG57, LEU55, and ARG67. Further, 2-amino-phenylamino-dibenzosuberone re-docked interacts with ASP168, ILE84, TYR35, GLY110, ALA111, VAL30, ALA51, VAL38, LEU167, LEU75, LYS53, and LEU104, of which conventional hydrogen bond interactions are formed with ASP168.

### 2.3. Molecular Docking Strategy Targeting IRAK-4

**Figure 5:**
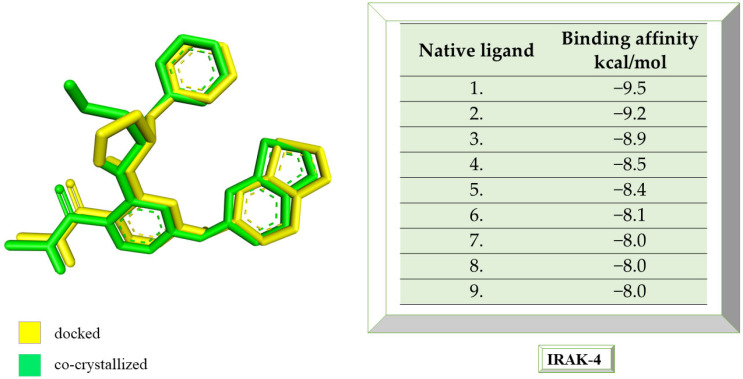
The highest binding affinity values for IRAK-4 of the re-docked native ligand, and the structure of the native ligand overlapped with the structure of the re-docked ligand. IRAK-4, interleukin-1 receptor-associated kinase 4.

IRAK-4 was co-crystallized with 6-[(1,3-benzothiazol-6-yl)amino]-4-{[(2S)-1-hydroxy-3-phenylpropan-2-yl]amino}-N-methylpyridine-3-carboxamide. The grid box size remains 60 × 60 × 60, and the coordinates have been set to X = 8.36, Y = 43.02, and Z = 9.82. Following the re-docking process, an RMSD value of 0.98 Å was obtained, indicating that the docking process went well. The most stable conformation shows an affinity of −9.5 kcal/mol towards IRAK-4. In Figure 5, the structure of the docked native ligand is overlaid with the structure of the co-crystallized ligand, and the affinities of the most stable conformations towards IRAK-4 are depicted.

The binding affinities of the ligands studied for their potential to interact with IRAK-4 are presented in Table 3.

**Figure 6:**
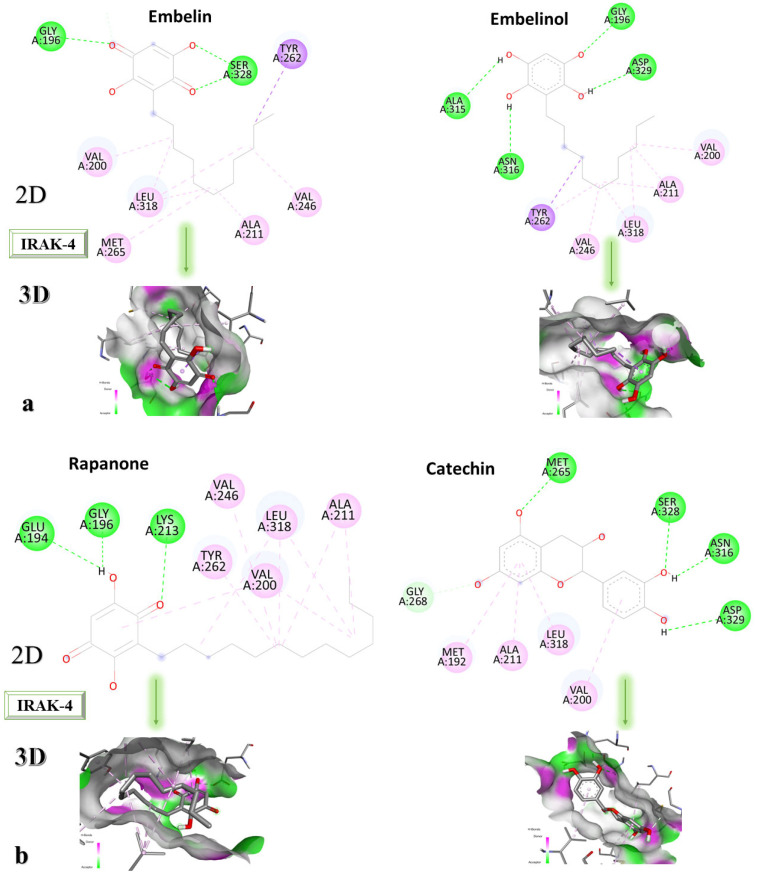

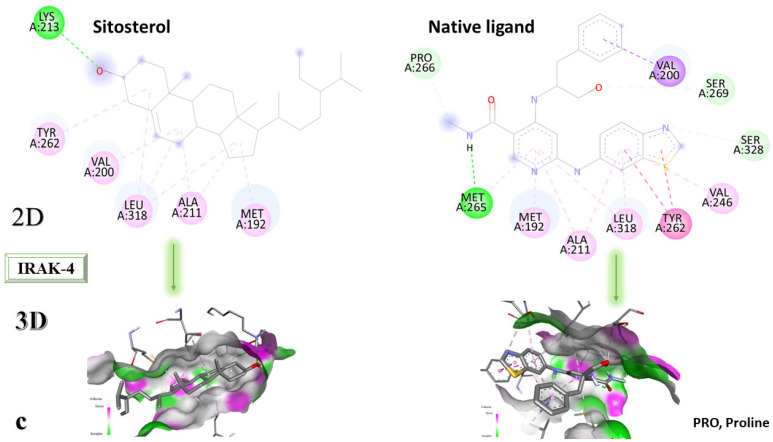
In-depth 2D and 3D interactions of ligands with different IRAK-4 constituent amino acids. IRAK-4, interleukin-1 receptor-associated kinase 4. (**a**). Interactions between the chemical structures of embelin and embelinol with the amino acids of IRAK-4; (**b**). Interactions between the chemical structures of rapanone and catechin with the amino acids of IRAK-4; (**c**). Interactions between the chemical structures of sitosterol and the native ligand with the amino acids of IRAK-4.

The compound showing the highest binding affinity to IRAK-4 is catechin, with −7.7 kcal/mol, followed by sitosterol, with −7.6 kcal/mol. The other 3 compounds show an affinity for proteins of −6.2 kcal/mol. Figure 6a–c shows the type of ligand-protein binding in both 2D and 3D formats.

Embelin interacts with TYR262, SER328, GLY196, VAL200, LEU318, MET265, ALA211, and VAL246 and forms conventional hydrogen bonds with GLY192 and SER328, according to the 2D diagram. Embelinol interacts with VAL200, ALA211, LEU318, VAL246, TYR262, ASN316, ALA315, GLY196, and ASP329, with which it forms conventional hydrogen bonds with ASN316, ALA315, GLY196, and ASP329. In the case of rapanone, it interacts with ALA211, LEU318, VAL200, TYR262, VAL246, LYS213, GLY196, and GLU194, forming conventional hydrogen bonds with the last three amino acids. The catechin-protein bond is achieved via the following amino acids: MET192, ALA21, LEU318, VAL200, ASP329, ASN316, SER328, and MET265, with the last 4 amino acids forming conventional hydrogen bonds. Sitosterol interacts with MET192, ALA211, LEU318, VAL200, TYR262, and LYS213, with which it forms conventional hydrogen bonds. The co-crystallized compound binds to VAL200, SER269, SER328, VAL246, TYR262, LEU318, ALA211, MET192, MET265, and PRO266, of which it forms conventional hydrogen bonds with MET265 and carbon-hydrogen bonds with SER328, SER269, and PRO266.

### 2.4. Molecular Docking Strategy Targeting MMP-9

**Figure 7:**
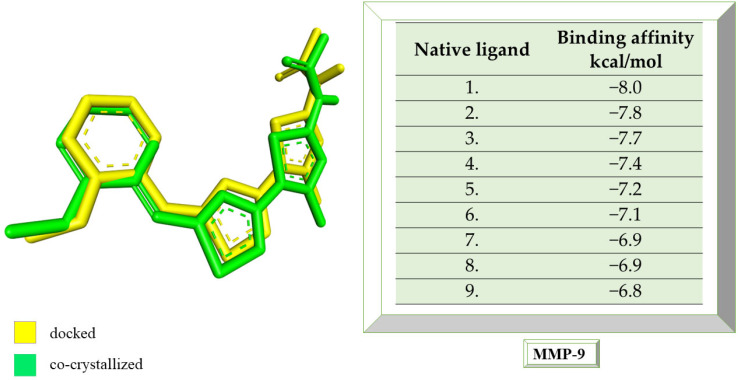
The highest binding affinity values for MMP-9 of the re-docked native ligand, and the structure of the native ligand overlapped with the structure of the re-docked ligand. MMP-9, matrix metallopeptidase 9.

MMP-9 was co-crystallized with the compound called JNJ0966 ([N]-[5-[2-[(2-methoxyphenyl) amino]-1,3-thiazol-4-yl]-4-methyl-1,3-thiazol-2-yl] ethanamide). Following the re-docking process, an RMSD of 1.32 Å was obtained. The grid box size remains 60 × 60 × 60, and the coordinates were set to X = 47.45, Y = 63.78, and Z = 42.03. Following the re-docking process, JNJ0966 showed an affinity of −8.0 kcal/mol towards MMP-9. In Figure 7, the structure of the docked native ligand is overlaid with the structure of the co-crystallized ligand, and the affinities of the most stable conformations towards MMP-9 are presented.

The binding affinities of the ligands studied for their potential to interact with MMP-9 are presented in Table 4.

**Figure 8:**
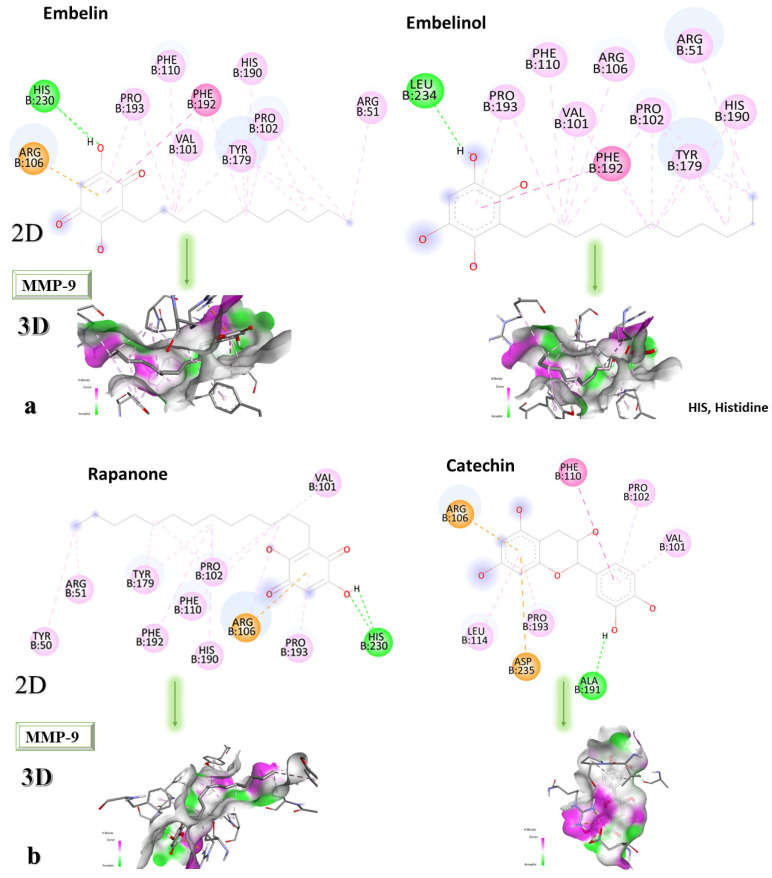

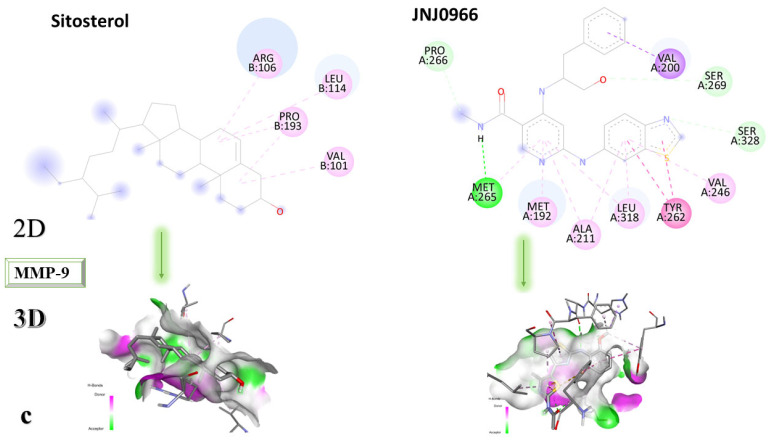
In-depth 2D and 3D interactions of ligands with different MMP-9 constituent amino acids. MMP-9, matrix metallopeptidase 9. (**a**). Interactions between the chemical structures of embelin and embelinol with the amino acids of MMP-9; (**b**). Interactions between the chemical structures of rapanone and catechin with the amino acids of MMP-9; (**c**). Interactions between the chemical structures of sitosterol and JNJ0966 with the amino acids of MMP-9.

Catechin remains the compound with the highest affinity, −8.0 kcal/mol, equal to that of the native compound JNJ0966. Sitosterol shows an affinity of −7.5 kcal/mol for the target protein, and embelin has an affinity of −7.3 kcal/mol. Embelinol shows the lowest affinity to MMP-9, with −6.9 kcal/mol. Figure 8a–c shows the type of binding that occurs between the ligand and the protein in both 2D and 3D formats.

The analysis of the 2D diagrams shows that embelin interacts with the following amino acids: ARG51, PRO102, TYR179, HIS190, PHE192, VAL101, PHE110, PRO193, HIS230 (conventional hydrogen bond), and ARG106. Embelinol interacts with HIS190, ARG51, TYR179, PRO102, PHE192, ARG106, VAL101, PHE110, PRO193, and LEU234. Rapanone interacts with HIS230 (conventional hydrogen bonds), PRO193, ARG106, HIS190, PRO102, PHE110, PHE192, TYR179, ARG51, TYR50, and VAL101. Catechin interacts with VAL101, PRO102, PHE110, ARG106, LEU114, ASP235, PRO193, and ALA191 (conventional hydrogen bonds). Sitosterol interacts with ARG106, LEU114, PRO193, and VAL101. JNJ0966 interacts with PHE110, HIS190, ALA191, VAL101, PHE192, TYR179, ARG106, LEU114, PRO193, and PRO102.

### 2.5. Virtual Screening of the Most Promising Candidates

Based on the structure of the compounds with the highest affinity to the studied proteins, (BTK, p38 MAPK, IRAK-4, and MMP-9), a ligand-based virtual screening study was performed using the online platform SwissSimilarity. The main goal was to identify compounds with similar chemical structures and to test them by molecular docking against the above-mentioned target proteins to identify compounds with therapeutic potential in RA, if associated with extensive studies, to determine safety and efficacy profiles.

SwissSimilarity compares chemicals and finds similarities using a variety of chemical fingerprinting techniques. Both 2D and 3D structural comparison approaches are used in these systems. SwissSimilarity offers information on the physicochemical characteristics of compounds, in addition to searching for similar compounds [37].

Chemical fingerprints of the extended-connectivity type (ECFPs) are frequently employed to represent and differentiate molecular structures. Since ECFPs are circular fingerprints, they contain data about the atoms in a molecule. By considering all feasible pathways between atoms in a molecule of a certain length (the fingerprint radius), the ECFP algorithm creates these fingerprints using a graph-based methodology. It has been demonstrated that ECFPs are highly used in a number of tasks, including similarity searching, virtual screening, and compound library clustering [38].

An advanced search for target compounds in the ZINC database (https://zinc.docking.org/ accessed on 24 April 2023) was conducted utilizing ECFPs. Based on the structure of sitosterol, the compound with the highest affinity for the target protein, a total of 150 compounds with similarity scores between 0.656 and 0.500 were identified. A query structure’s similarity to a collection of reference compounds known to possess activity against a target protein or pathway is measured by the similarity score.

The chemical structure, affinity, and similarity score for the 5 most promising compounds in terms of affinity to BTK are presented in Table 5.

ZINC000004024651 showed the highest affinity for BTK and may be a potential candidate for BTK inhibition.

In the case of p38 MAPK, embelin was selected for virtual screening because it showed the highest affinity for the target protein. Following the methodology presented above, 140 compounds were identified with similarity scores, ranging from 0.674 to 0.300. The chemical structure, affinity, and similarity score for the 5 most promising candidates, in terms of protein affinity, are presented in Table 6.

The compound named ZINC000000434197 shows the highest affinity to the target protein with −10.0 kcal/mol, thus having a higher affinity than the parent compound (embelin, −8.4 kcal/mol), but a lower affinity than the native protein compound (−11.1 kcal/mol).

In the case of IRAK-4, the process is similar, but the compound that showed the highest affinity to the protein is called catechin, which shows an affinity of −7.7 kcal/mol, compared to the −9.5 kcal/mol of the parent compound. The chemical structure, affinity, and similarity score for the 5 most promising compounds, in terms of affinity for IRAK-4, are presented in Table 7.

Following ligand-based virtual screening docking of the compounds, ZINC000087606977 is found to have the highest affinity to the target protein (−10 kcal/mol), outperforming both the re-docked native ligand (−9.5 kcal/mol) and the parent compound (catechin with −7.7 kcal/mol).

For the last studied MMP-9 protein, the compound showing the highest affinity is catechin. Both the native compound and the docked compound show identical affinities to the protein (−8.0 kcal/mol). The chemical structure, affinity, and similarity score for the top 5 compounds, in terms of affinity to proteins, are shown in Table 8.

Of the compounds selected from the ZINC database, ZINC000014728393 exhibited the highest affinity for the target protein (−8.7 kcal/mol) and had a higher affinity for the protein than both the parent compound and the native ligand. This result indicates that it may be a promising ligand, requiring further investigation to determine its efficacy in RA.

**Figure 9:**
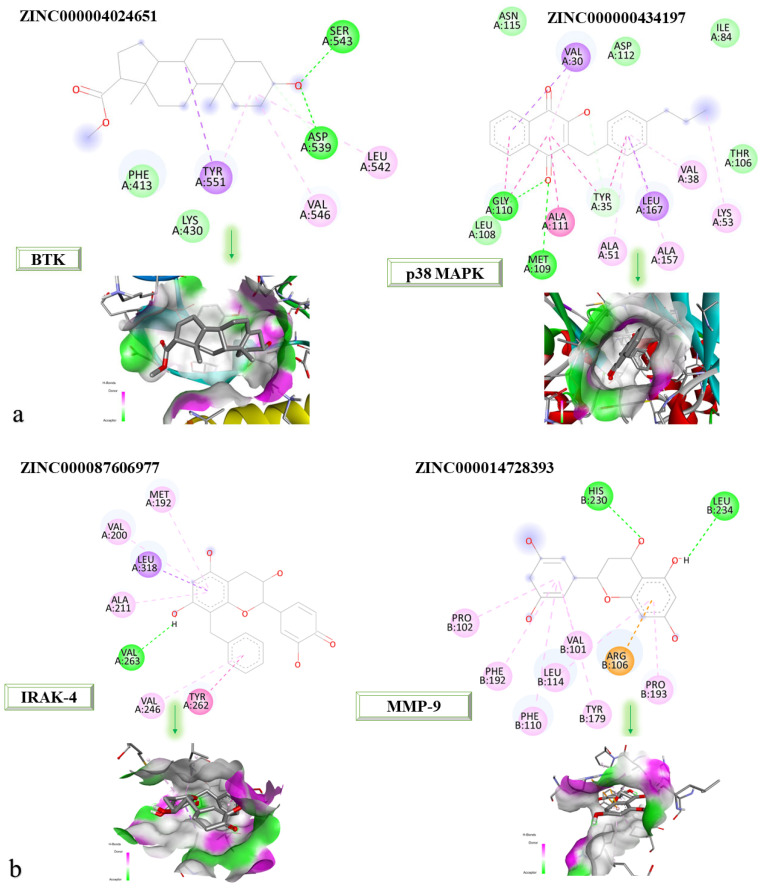
In-depth 2D and 3D interactions of the most promising newly identified ligands with different constituent amino acids of target proteins. (**a**). The interactions between the chemical structures of ZINC000004024651with the amino acids of BTK and the interactions between the chemical structures of ZINC00000434197 with the amino acids of p38 MAPK; (**b**). The interactions between the chemical structures of ZINC0000087606977 with the amino acids of IRAK-4 and the interactions between the chemical structures of ZINC0000014728393 with the amino acids of MMP-9.

Figure 9a,b shows the 2D and 3D structures at the protein-binding site of the newly identified compounds that were found to be the most promising following the molecular docking process.

According to the 2D diagram, it can be observed that ZINC000004024651 interacts with the following amino acids: SER543, ASP539, LEU542, VAL546, and TYR551. The amino acid with which both the parent compound and ZINC000004024651 interact is LEU542. ZINC000000434197 interacts with the following amino acids: VAL30, GLY110, MET109, ALA111, TYR35, ALA51, LEU167, ALA157, VAL38, and LYS53, and the amino acids common to the parent compound are listed as: ALA51, LEU167, MET109, ALA157, VAL30, VAL38, LYS52, and TYR35. ZINC000087606977 interacts with the following proteins: MET192, VAL200, LEU318, ALA211, VAL263, VAL246, and TYR262. All the amino acids with which this compound interacts are also found in the native compound, resulting in a similar binding site for both the native and parent compounds. ZINC000014728393 showed the highest affinity for MMP-9. It interacts with the following amino acids: LEU234, HIS230, PRO102, PHE192, PHE110, LEU114, VAL101, TYR179, ARG106, and PRO193, of which those common to the parent compound are PRO102, PHE192, PHE110, LEU114, VAL101, TYR179, ARG106, and PRO193.

The four newly identified compounds have the potential to be explored as adjuvant therapies for the treatment of RA. It is important to mention that this is only a preliminary stage of in silico studies that requires further experimental endorsement based on in vitro and in vivo studies. With its high affinity for BTK, ZINC000004024651 could be investigated as a potential adjuvant therapy, alongside existing B cell-targeted treatments, leading to improved control of the autoimmune response and a reduction in inflammation in RA [33]. Due to its increased affinity for p38 MAPK, ZINC000000434197 could be considered a potential adjuvant therapy, in conjunction with conventional RA treatments. ZINC000000434197 may provide additional anti-inflammatory effects and enhance disease control in RA patients by specifically targeting the production of pro-inflammatory cytokines mediated by p38 MAPK [35]. With its higher affinity for IRAK-4, ZINC000087606977 has the potential to be used as an adjuvant therapy for modulating the inflammatory signaling pathways associated with Toll-like receptors and IL-1 receptors [34]. ZINC000014728393, displaying a high affinity for MMP-9, could be investigated as a potential adjuvant therapy to mitigate joint tissue degradation in RA. Moreover, by inhibiting MMP-9, ZINC000014728393 may help preserve joint integrity and delay the development of structural damage, when used in conjunction with existing therapies [32].

### 2.6. In Silico Evaluations of the Pharmacokinetic Profile of Novel Identified Compounds

The SwissADME (http://www.swissadme.ch/ accessed on 28 April 2023) platform provides information related to lipophilicity, absorption, metabolism, and solubility of newly identified compounds by ligand-based virtual screening. Table 9 displays the results with pharmacokinetic implications after analyzing the information input into the platform.

The molecular masses of the four newly identified compounds are below 500 g/mol, thus falling into the category of small molecules for which SwissADME can make pharmacokinetic estimates. All four compounds adhere to Lipinski’s principles (i.e., molecular weight less than 500 g/mol, maximum 5 H-bond donors, maximum 10 H-bond acceptors, and a LogP value of maximum 5), implying that oral administration may be possible in the future, if extensive studies on their efficacy and safety profile confirm [39].

The compound ZINC000004024651, identified as a potential BTK inhibitor, has a molecular weight of 346.50 g/mol, similar to that of approved BTK inhibitors such as Ibrutinib, Acalabrutinib, and Zanubrutinib, which have molecular weights of 440.5 g/mol, 465.5 g/mol, and 471.5 g/mol, respectively. Additionally, the compounds ZINC000000434197 (306.36 g/mol), ZINC000087606977 (380.39 g/mol), and ZINC000014728393 (288.25 g/mol), identified as potential inhibitors of p38 MAPK, IRAK-4, and MMP-9, respectively, have molecular masses that adhere to Lipinski’s rule regarding molecular weight. Additionally, approved JAK inhibitors for managing RA, including Baricitinib (371.4 g/mol), Tofacitinib (312.37 g/mol), and Upadacitinib (380.4 g/mol), also possess molecular masses within the range that respect Lipinski’s rule. Based on the molecular weight, we can estimate some of the properties of the compounds. Molecular weight usually influences the solubility of the compounds, with compounds that have a high molecular weight tending to be more lipophilic. The molecular weight can also affect the absorption of the compound, with smaller molecules tending to diffuse through membranes more easily. Based on this data, we may estimate that the molecules we identified could behave similarly to known JAK inhibitors, but it is important to note that the molecular weight is just one of the factors that influence the potential effectiveness of the compounds [40].

The major differences between the four compounds are noted in the potential inhibitory roles of the CYP450 isoforms; thus, the evaluation of their metabolism is becoming essential. Inhibitory action may lead to drug interactions and toxicity phenomena, or may decrease the effect if the co-administered molecule is a prodrug that is activated at the level of that enzymatic pathway [41].

It is important to highlight that the Lipinski principle is merely a recommendation that can assist researchers in deciding which substances to focus their further research on. It does not accurately predict a compound’s pharmacokinetic characteristics.

SwissADME can evaluate solubility, lipophilicity, permeability, and pharmacokinetics using computational models and algorithms. In silico approaches to examining drug ADME properties can be valuable in early drug design, but they do not substitute for in vivo examinations. Instead, they offer an economical and rapid approach to assessing small-molecule pharmacokinetic characteristics [42].

Starting from phytocompounds with anti-inflammatory and antioxidant potential identified in various parts of *Embelia ribes*, molecular docking studies evaluating interactions with target proteins involved in the pathophysiology of RA and ligand-based virtual screening have provided a series of compounds with affinities for target proteins that may represent future solutions in the management of RA, but require further validation.

The following aspects represent the strengths of the current study: estimation of the binding affinity and interaction mode between a protein target and a ligand with demonstrated anti-inflammatory effects, which can direct the configuration of novel ligands; screening of a sizable database of compounds for potential lead molecules; provision of a thorough analysis of the physicochemical and pharmacokinetic properties of newly identified molecules; and highlighting the potential of phytocompounds to lead to new compounds that, when optimized, can contribute, through further evaluation, to RA management.

The present study has several limitations, such as the inability to predict the biological activity of a molecule and the focus on a single static interaction between the ligand and the protein of interest, even though protein molecules are highly mobile in the biological environment and can substantially change their conformation throughout compound binding. Another factor that may affect ligand binding is the incapacity to evaluate the solvent effect. These limitations appear in this first stage of the study, but can be managed by future research directions, including newer computational methods (i.e., molecular dynamics simulation, network pharmacology, fragment-based docking, etc.). However, all the results obtained will need further experimental validation for going through all the necessary steps regarding their approval in therapy.

## 3. Materials and Methods

### 3.1. Ligand Preparation

The final selection of ligands was made based on previously collected experimental data, in which known binders to the target of interest were chosen. Based on scientific literature studies, and taking into consideration experimental assays and the algorithms for in silico evaluations, five bio compounds (i.e., embelin [43], embelinol [16], rapanone [44], catechin [45], and sitosterol [46]) with anti-inflammatory and antioxidant potential were selected for the present study, all of them being contained in different parts of *Embelia ribes*. The five phytoconstituents were processed in Simulation Description Format (.SDF) using the PubChem database (https://pubchem.ncbi.nlm.nih.gov/, accessed on 15 April 2023). Because the .SDF file format is incompatible with the docking software (AutoDock Tools 1.5.7, https://vina.scripps.edu/, accessed on 20 April 2023) [47], these files were transformed into the Autodock-specific format (Protein Data Bank, Partial Charge (Q) and Atom Type (T) format) using the Open Babel GUI 2.3.1 software (https://openbabel.org/docs/current/GUI/GUI.html/, accessed on 20 April 2023) [48]. Ligands were imported into the AutoDockTools 1.5.7 using the ligand input function to add assignments, as well as to determine rotatable bonds.

### 3.2. Protein Preparation

Target proteins selected based on their involvement in various pathophysiological pathways of RA identified in the medical literature were downloaded from the Research Collaboratory for Structural Bioinformatics PDB (https://www.rcsb.org/, accessed on 15 April 2023). Proteins with the following PDB codes were selected for this study: 5UE4-MMP-9, 3GEN-BTK, 5W85-IRAK-4, and 3ZYA-p38 MAPK. Molecular docking studies per se were performed using a ligand-binding protein and co-crystallized water. To remove the ligand and co-crystallized water, the Molegro Molecular Viewer software (http://molexus.io/molegro-molecular-viewer/, accessed on 15 April 2023) was used. Using the AutoDockTools 1.5.7, polar hydrogens and Gasteiger charges were added.

### 3.3. Molecular Docking Analysis

The molecular docking experiments were carried out using AutoDockTools, a widely used software program in the field of bioinformatics. Discovery Studio Visualizer 4.5 was used to generate the 2D and 3D images that provide a comprehensive visualization of the molecular docking results (i.e., protein-ligand interactions and spatial arrangement of the docked ligands) (https://www.3ds.com/products-services/biovia/products/molecular-modeling-simulation/biovia-discovery-studio/visualization/ accessed on 29 April 2023). For all the molecular docking calculations, the grid box size was set to 60 × 60 × 60 Å. The grid box was used to establish the space where the docking software explores possible poses. Prior to docking the investigated ligands, a validation procedure was employed that consisted of re-docking the native ligand of the investigated protein (the ligand that comes co-crystalized with the protein), followed by a comparison of the docked pose to the native pose. The RMSD determines the similarity between the atomic coordinates of the two positions and is calculated by comparing the native ligand to the re-docked ligand. The consensus is that molecular docking methods that generate docking poses with a RMSD value that is under 2 Å (lower values indicate a better match) are generally good at predicting ligand poses. In the presented study, all the docking poses of the re-docked native ligand had an RMSD under 2 Å, calculated using the AutoDockTools 1.5.7 software.

### 3.4. Virtual Screening

To identify new compounds with potential in the treatment of rheumatoid arthritis, the chemical structures of the compounds that showed the highest affinity for the proteins studied in molecular docking studies were used. Based on their structure, the SwissSimilarity (http://www.swisssimilarity.ch/ accessed on 24 April 2023) online platform was used to find compounds with similar chemical structures. The newly identified ligands were downloaded from the ZINC database in .SDF format and converted with OpenBabelGUI [48] into a format compatible with AutoDock Vina (https://vina.scripps.edu/, accessed on 20 April 2023) [47], where the necessary charges were added and rotatable bonds were determined. The next step of the study was to dock them to the target proteins to identify compounds with high binding affinity and a potential for further introduction into extensive studies to determine safety and efficacy profiles.

### 3.5. Pharmacokinetic Profile Assessments

The SwissADME (http://www.swissadme.ch/, accessed on 28 April 2023) online platform was applied to estimate the pharmacokinetic properties of molecules that were determined to be the most promising through molecular docking and ligand-based virtual screening analyses.

## 4. Conclusions

Phytocompounds from *Embelia ribes* may be used to treat RA and other diseases. Phytoconstituents in *Embelia ribes* can potentially target RA pathogenic proteins, making them suitable ligands for molecular docking, virtual screening, and pharmacokinetic studies. The current findings indicate the four new compounds that will require extensive experimental validation before their safety and efficacy profiles can be established, and may be used as adjuvants in the treatment of RA.

## Figures and Tables

**Table 1:** Docking score of the evaluated ligands.

Ligands	Binding Affinity (kcal/mol)	Chemical Structure
Embelin	−7.3	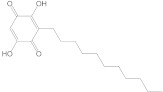
Embelinol	−7.1	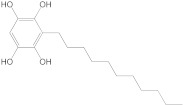
Rapanone	−6.8	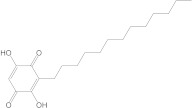
Catechin	−8.4	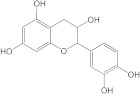
Sitosterol	−8.5	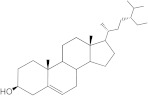

**Table 2:** Docking scores of the compounds investigated.

Ligands	Binding Affinity (kcal/mol)
Embelin	−8.4
Embelinol	−7.7
Rapanone	−7.9
Catechin	−8.3
Sitosterol	−8.2

**Table 3:** Docking scores of the compounds investigated.

Ligands	Binding Affinity (kcal/mol)
Embelin	−6.2
Embelinol	−6.2
Rapanone	−6.2
Catechin	−7.7
Sitosterol	−7.6

**Table 4:** Docking scores of the compounds investigated.

Ligands	Binding Affinity (kcal/mol)
Embelin	−7.3
Embelinol	−6.9
Rapanone	−7.2
Catechin	−8.0
Sitosterol	−7.5

**Table 5:** The 5 most promising compounds with affinity for BTK identified in the ZINC database.

Compound	Structure	Binding Affinity (kcal/mol)	Similarity Score
ZINC000004024651	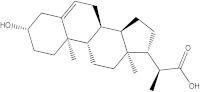	−8.6	0.586
ZINC000253607818	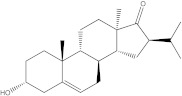	−8.5	0.522
ZINC000245240779	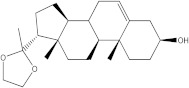	−8.4	0.514
ZINC000257349551	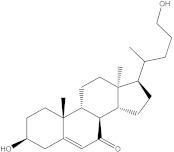	−8.3	0.506
ZINC000118931309	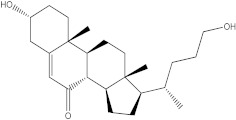	−8.3	0.506

**Table 6:** The 5 most promising compounds with affinity for p38 MAPK, identified in the ZINC database.

Compound	Structure	Binding Affinity (kcal/mol)	Similarity Score
ZINC000000434197	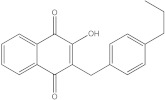	−10.0	0.346
ZINC000104240396	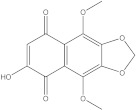	−8.6	0.353
ZINC000005513529	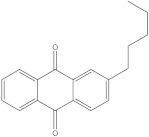	−8.4	0.354
ZINC000104240396	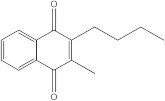	−8.3	0.353
ZINC000000434197	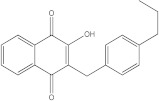	8.3	0.346

**Table 7:** The 5 most promising compounds with affinity for IRAK-4, identified in the ZINC database.

Compound	Structure	Affinity (kcal/mol)	Similarity Score
ZINC000087606977	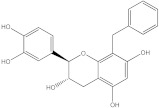	−10.0	0.519
ZINC000000004935	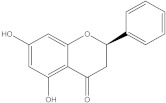	−9.1	0.389
ZINC000014819291	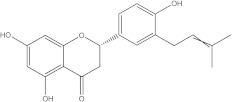	−8.9	0.354
ZINC000000001785	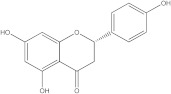	−8.6	0.423
ZINC000003022616	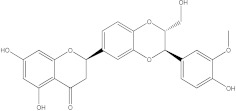	−8.6	0.356

**Table 8:** The 5 most promising compounds with affinity for MMP-9, identified in the ZINC database.

Compound	Structure	Affinity (kcal/mol)	Similarity Score
ZINC000014728393	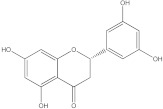	−8.7	0.385
ZINC000014766771	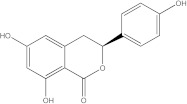	−8.6	0.377
ZINC000000058117	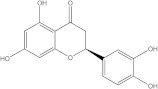	−8.5	0.50
ZINC000014642733	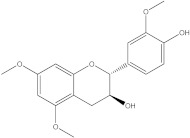	−8.2	0.509
ZINC000014819291	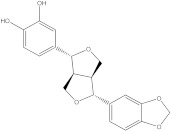	−8.1	0.354

**Table 9:** Pharmacokinetic data obtained from the newly identified compounds.

Characteristics	ZINC000004024651 (BTK)	ZINC000000434197 (p38 MAPK)	ZINC000087606977 (IRAK-4)	ZINC000014728393 (MMP-9)
Formula	C_22_H_34_O_3_	C_20_H_18_O_3_	C_22_H_20_O_6_	C_15_H_12_O_6_
Molecular weight	346.50 g/mol	306.36 g/mol	380.39 g/mol	288.25 g/mol
Num. rotatable bonds	2	4	3	1
Num. H-bond acceptors	3	3	6	6
Num. H-bond donors	2	1	5	4
Log P	3.05	2.97	2.25	1.52
Gastrointestinal absorption	High	High	High	High
CYP2C19 inhibitor	No	Yes	No	No
CYP2C9 inhibitor	Yes	Yes	No	No
CYP2D6 inhibitor	No	Yes	Yes	No
CYP3A4 inhibitor	No	Yes	Yes	Yes
Lipinski	Yes	Yes	Yes	Yes

## Data Availability

Information provided in this research are supported by the inserted references or have been generated by using the softs mentioned in the main text.
